# Alleviating the concerns with the SDT approach to reasoning: reply to Singmann and Kellen (2014)

**DOI:** 10.3389/fpsyg.2015.00184

**Published:** 2015-02-19

**Authors:** Dries Trippas, Michael F. Verde, Simon J. Handley

**Affiliations:** ^1^Center for Adaptive Rationality, Max Planck Institute for Human DevelopmentBerlin, Germany; ^2^School of Psychology, Cognition Institute, Plymouth UniversityPlymouth, UK

**Keywords:** reasoning, signal detection theory, model fitting, model identifiability, belief bias, response bias

In their comment on our article (Trippas et al., 2014a), Singmann and Kellen (2014; henceforth SK) suggest that our use of signal detection theory (SDT) provides an uninformative characterization of the data, that our application of SDT to causal-conditional reasoning is unnecessary and misguided, and that the model does not provide a good fit of the data. We will address each of these points.

SK's concern that our use of SDT is uninformative rests on a single issue: how to interpret the shift in the location of the confidence points along the ROC when comparing the believable and unbelievable conditions (Trippas et al., 2014a; Figure 1), a shift that in real terms represents a greater tendency to accept believable arguments as “valid.” We find SK's focus on this aspect of the data surprising because it has no direct bearing on the main points of the article, which have to do with the changes in the shape and separation of the ROCs when we segregate the data in different ways (Trippas et al., 2014a, Figure 1, comparing the top and bottom panels). For this reason, we mentioned the confidence point shift only once, saying that it fits a pattern previously interpreted by Dube et al. (2010) as a shift in response bias, but which might also be due to a symmetric shift in the evidence distributions. This is a succinct way of stating what SK describe in great detail in their toy model. We have no problem with the fact that the confidence point shift has alternative interpretations. Although it is not integral to the thrust of the article, this aspect of the data is worth noting because the pattern is observed in other reasoning tasks and represents a point of continuity despite the apparent discontinuity in other aspects of the ROC data.

We gather that SK's focus on the unidentifiability issue is meant to be a critique of the SDT model in general. In our view, it is not a compelling critique. Following convention, we use “accuracy” to denote sensitivity, the ability to discriminate classes of items (valid from invalid). Accuracy depends on the relative distance between the valid and invalid distributions. If some factor were to increase the argument strength of invalid and valid arguments by exactly the same degree, accuracy would remain constant. This is a specific circumstance which the SDT model cannot distinguish from a shift in response bias. The model is, however, unambiguous in distinguishing between changes in accuracy from those that might be ascribed solely to response bias. This is where the theoretical power of the model lies (e.g., Trippas et al., 2013).

As for the question of response bias, the SDT model is widely used in domains like memory where criterion placement is an issue because theorists have a range of other tools at their disposal to deal with ambiguity (they can, for example, use manipulations that plausibly only affect response bias). In their final point, SK cite work in recognition memory (Morrell et al., 2002) to argue that trial-by-trial criterion shifts are implausible. These findings describe the specific case in which test stimuli are indistinguishable save for an internal signal of mnemonic strength. When the stimuli are overtly distinguishable on other dimensions, people seem quite capable of shifting their criterion from one trial to the next (Dobbins and Kroll, 2005; Aminoff et al., 2012). Whether people do use different response criteria when judging believable and unbelievable arguments remains an open question, but the memory literature provides ample reason to believe that it is plausible.

SK argue against the application of SDT to conditional reasoning because one can reach the same conclusions by examining raw acceptance rates. This misses the point of using a model like SDT, which is to view the data within a consistent, theoretically justified framework. The problems that can arise when raw acceptance rates are used to measure accuracy are well documented (Klauer et al., 2000; Dube et al., 2010; Heit and Rotello, 2014) and certainly apply here.

SK make a good point in observing that the fit of the model to Roser and colleagues' ROC curves is poor. The problem may lie in the application of the model to aggregated data. It is well known that *G*^2^ depends on sample size such that aggregate model fits very often lead to violations of absolute fit. One alternative approach is to evaluate model fit for each participant individually (Cohen et al., 2008). To demonstrate the problematic nature of assessing the model fit of aggregated samples in terms of *G*^2^ when sample sizes are large, we combined data from 131 participants from previously published work on belief bias (Trippas et al., 2013, 2014b). We fit the believable and unbelievable ROCs separately, both aggregated and on a per-participant basis. The aggregate fit to the believable ROC was borderline acceptable, *G*^2^_(3)_ = 6.97, *p* = 0.07. For the unbelievable ROC, the fit was unacceptable, *G*^2^_(3)_ = 50.6, *p* < 0.001. The individual fits paint a prettier picture: for the believable problems, only 9 out of 131 or less than 7% of the participants show a violation of fit (*p* < 0.05). The unbelievable-problems case fares even better, with only 4 out of 131 or about 3% of the participants producing ill-fitting data patterns. How can such drastically different patterns of fit emerge? Individual differences potentially play a large role: unbelievable problems elicit different reasoning strategies in different people (Trippas et al., 2013, 2014b,c), and aggregating across such data patterns will suggest that the model is inappropriate. We suspect that similar factors have contributed to the poor fits reported by SK.

SK's comments speak to a number of interesting issues that deserve to be raised in the wider discussion surrounding the SDT approach to modeling human reasoning. It is useful, however, to reiterate the point of our original article which seems to be lost in the discussion of side issues. A strict adherence to “normativism” often leads investigators to biased or misleading interpretations of phenomena (Elqayam and Evans, 2011). The default normative approach to the application of SDT to reasoning illustrates precisely this problem in the case of causal conditionals.

## Conflict of interest statement

The authors declare that the research was conducted in the absence of any commercial or financial relationships that could be construed as a potential conflict of interest.
